# An evaluation of the effects of saffron supplementation on the asthma clinical symptoms and asthma severity in patients with mild and moderate persistent allergic asthma: a double-blind, randomized placebo-controlled trial

**DOI:** 10.1186/s12931-019-0998-x

**Published:** 2019-02-22

**Authors:** Marzie Zilaee, Seyed Ahmad Hosseini, Sima Jafarirad, Farhad Abolnezhadian, Bahman Cheraghian, Foroogh Namjoyan, Ataollah Ghadiri

**Affiliations:** 1 Nutrition & Metabolic Diseases Research Center, Ahvaz Jundishapur University of Medical Sciences, Ahvaz, Iran; 2 Nutrition Department, Faculty of Paramedicine, Ahvaz Jundishapur University of Medical Sciences, Ahvaz, Iran; 3 Division of Immunology and Allergy, Department of Pediatrics, Abuzar Children’s Hospital, Ahvaz Jundishapur University of Medical Sciences, Ahvaz, Iran; 4 Research Center for Infectious Diseases of Digestive System, Department of Biostatistics and Epidemiology, School of Public Health, Ahvaz Jundishapur University of Medical Sciences, Ahvaz, Iran; 5 Pharmacognosy Department, Marine Pharmaceutical Research Center, Faculty of Pharmacy, Ahvaz Jundishapur University of Medical Sciences, Ahvaz, Iran; 6 Department of Immunology, School of Medicine, Ahvaz Jundishapur University of Medical Sciences, Ahvaz, Iran

**Keywords:** Asthma severity, Allergic asthma, Saffron

## Abstract

**Background:**

Asthma is a heterogeneous disease which is usually associated with chronic airway inflammation. Saffron has anti-inflammatory effects and may has beneficial effects on asthma.

**Hypothesis:**

The present study was intended to survey the effects of saffron supplementation on blood pressure, lipid profiles, basophils, eosinophils and clinical symptoms in patients with allergic asthma.

**Study design:**

Our study was a clinical trial.

**Methods:**

Subjects (*N* = 80, 32 women and 48 men, 41.25 ± 9.87 years old) with mild and moderate allergic asthma were randomized into two groups: the intervention group who received two capsules of saffron (100 mg/d), and the control group who received two capsules of placebo for 8 weeks. SPSS software (version 16.0) was used for the data analysis.

**Results:**

Saffron improved the frequency of clinical symptoms of the patients (i.e., frequency of the shortness of breath during the day and night time, use of salbutamol spray, waking up due to asthma symptoms and activity limitation) in comparison to the placebo (*p* < 0.001). Besides, asthma severity decreased almost significantly in the saffron group (*p* = 0.07). It was also found that saffron, in comparison with the placebo, significantly reduced the systolic and diastolic blood pressure, triglycerides and low density lipoprotein cholesterol. Moreover, eosinophils and basophils concentration reduced in the saffron group (*p* = 0.06 and 0.05 respectively).

**Conclusion:**

Saffron seems to be an effective and safe option (in 8 weeks supplementation) to improve clinical symptoms of patients with allergic asthma but the toxicity and/or long-term effects of saffron intake are not known. Registration ID in IRCT (IRCT2017012132081N2).

## Key points

- There is some evidence supporting the role of inflammation in the pathogenesis of allergic asthma. According to previous studies, saffron has been found to have anti-inflammatory effects.
- Saffron was found to be effective in alleviating clinical pulmonary symptoms in allergic asthmatic patients. Moreover, asthma severity improved following saffron supplementation.
- This study implies that saffron may have beneficial effects on allergic asthma.

## Backgrounds

Asthma, as a heterogeneous disease is usually associated with chronic airway inflammation. It is defined by the history of respiratory symptoms such as wheeze, chest tightness, shortness of breath and cough that vary over time and in intensity, together with variable limitations in expiratory airflow. Allergic asthma is the most easily recognized phenotype of asthma which often starts in childhood [1].It is estimated that 300 million people worldwide suffer from asthma, and this number is expected to increase to 400 million by 2025. The global prevalence of asthma diagnosed in adults was estimated to be 4.3% [1]. In turn, annual worldwide deaths from asthma have been estimated at 250,000 people [2]. The prevalence of asthma in Iran is reported to be about 5.5% [3]. The WHO has estimated that 15 million disability-adjusted life years due to asthma are lost annually [4]. Asthma is related to a past or family history of allergic disease such as eczema, food or drug allergy or allergic rhinitis. Asthmatic patients usually respond well to inhaled corticosteroid (ICS) treatment [2]. In the process of airway inflammation, a variety of immunologic cells, such as mast cells, eosinophils, lymphocytes, and neutrophils are involved [5]. Eosinophils accumulate preferentially at sites of allergic inflammation such as allergic asthma and release a variety of inflammatory mediators including radical oxygen species and cytokines which they can play an important role in the pathophysiology of asthma [6].

One of the risk factors of asthma isgenetic. andSome triggers of asthma symptoms which are usually associated with environmental factors include exposure to allergens and access to healthcare services [2].It is believed that should individuals, healthcare providers and organizations, local and national governments and healthcare organizations work collectively to improve asthma control [2].

In traditional medicine, saffron *(Crocus sativus L.)* has been used for the treatment of heart disease, depression, stress and sleep disorders [7]. Saffron has antioxidant [8], anti-inflammatory [9] and muscle relaxant effects [10]. Active components of saffron such as safranal and crocin also have anti-inflammatory and antioxidant effects and so may have beneficial effects on asthma [7]. It is reported that saffron in animals with allergic asthma reduced their total white blood cells, eosinophils and basophils, as some of these effects were found to be equal to dexamethasone [7]. Besides, saffron supplementation in guinea pig with allergic asthma reduced the serum level of endothelin1 (as an inflammatory index) [11]. Results of the study of Boskabady et al., showed a potent relaxant effect of saffron on tracheal chains of guinea-pigs which was comparable to or even higher than that of theophylline [10]. In turn, crocin (one of the active components of saffron) supplementation was reported to decrease low density lipoprotein cholesterol (LDL-C), total cholesterol and triglyceride in rats with type 2 diabetes mellitus [12].

According to the anti-inflammatory and antioxidant activity of saffron, it seems that saffron supplementation can beneficially affect asthmatic patients. Thus the present study, which is to our best knowledge the first clinical trial in the relationship between asthma and saffron, investigated the effect of saffron supplementation on asthma clinical symptoms, asthma severity score, blood pressure and lipid profiles in patients with mild and moderate persistent allergic asthma in arandomized clinical trial.

## Materials and method

### Trial design

This was a randomized, double blind, and placebo-controlled clinical trial with a parallel design. The sample size was calculated based on the comparison of ratios formula with α = 0.05 and β = 0.2. As there is no similar study to our trial, we used the data of our pilot study and by р_1_ = 0.231 and p_2_ = 0.0, the sample size of 29 subjects in each group was calculated. Considering the probability of filling 25% of the samples, the final sample size of 39 subjects in each group (78 subjects) was calculated. The sample size calculation was based on the asthma severity change.

The comparison of ratios formula $n=\frac{{\left({Z}_{1-a/2}+{Z}_{1-\beta}\right)}^{2\ast}\left[{p}_1\left(1-{p}_1\right)+{p}_2\left(1-{p}_2\right)\right]}{{\left({p}_{1-}{p}_2\right)}^2}$

Considering the length of the treatment, 40 subjects were finally enrolled in each group (1: 1 ratio) according to a computer-generated blocked randomization list with a block size of 6. To decrease the probable bias, we used an allocation concealment technique. A researcher, who did not have any clinical participation in the study placed the saffron and placebo capsules into numbered bottles based on a random list. Another researcher, who was not aware of the random sequences and was not involved in the trial, assigned the numbered bottles to the patients. After the allocation was done, the participants were asked to fill in all the questionnaires during the baseline visit. The blood samples were then collected in the next day and the participants were accordingly allowed to start having treatment capsules. Filling the questionnaires and taking blood samples were repeated again at the end of the study. Every two weeks all the participants were contacted to remind them about taking the treatment capsules and also ask them to report possible side effects of the treatment. Anthropometric measurements including weight and height were determined pre- and post-intervention. Disease history, demographic data, history of using medications and supplements, history of smoking and physical activity level (assessed by the short form of the International Physical Activity Questionnaire) were also assessed. All the participants, researchers, and the physicians were blind to the allocations using the random codes until the statistical analyses were completed. In the eight-week study period, all the participants were given dietary advices such as avoiding consumption of saffron (from food), fast foods, sausage, smoked and canned foods. Patients were also asked not to change their energy intake and physical activity during the study. To control the energy intake, we used the 24-h recalls of 3 days (2 work days and one weekend day). According to the doctor recommendations, patients were asked not to go out when the air was polluted and use filter masks in emergencies. The lung specialist used the same drugs for the patients in this study. Compliance was monitored during a weekly call, assessing compliance by asking the number of capsules consumed. Those subjects who did not take their capsules regularly or were intolerant to the medication were excluded from the study. Our patients had no disease except asthma and consumed no drug except asthma drugs. Personal information of participants collected, shared and maintained in order to protect confidentiality before, during, and after the trial by coding the patients.

### Participants

Participants included 80 men and women between the ages of 18 and 65 years. According to the Global Initiative for Asthma (GINA) diagnostic criteria for asthma, 80 patients with mild and moderate allergic asthma were examined by an attendant pulmonologist in May and October 2017, and were then recruited from the outpatient clinic at Imam Khomeini Hospital, Ahvaz, Iran. After that, the participants were provided with information about the study by both verbal explanation and written information sheets. They were asked to take the capsules regularly, provide adequate tracking information, and complete the questionnaires and the treatment procedure until the end of the study. To collect information on their socio-demographic status, occupation, smoking behavior, medical history and medication, all patients were also interviewed. The inclusion criteria were an age range of 18–65 years, with mild and moderate persistent allergic asthma according to the GINA criteria and body mass index (BMI) up to 27 kg/m^2^. The exclusion criteria included smoking, pregnancy, lactation, diabetes, autoimmune disease, malignancy and other pulmonary diseases. The chemical drugs which our patients consumed were not significantly different between saffron and placebo groups (*p* = 0.66). All the patients provided written informed consent, and the whole protocol met the requirements of Ahvaz Jundishapur University of Medical Sciences Ethics Committee.

### Intervention

During the intervention, all the patients received either two oral capsules including 50 mg dries saffron *(Crocus sativus L.)* stigma or a placebo (all the capsules were made in the Faculty of Pharmacy at Ahvaz Jundishapure University of Medical Sciences). The placebo capsules contained edible colors similar in color, shape, size, and package to the saffron ones. Dried saffron stigma was taken from Estahban, Fars province, Iran (Herbarium code: JPS018118). It was formulated as a capsule containing 50 mg of dried saffron stigma and starch (as fillers). The placebo capsules contained starch and permitted food colors to trace amount (E104, E110 and E122). The patients received the capsules at the start of the trial in the pulmonary clinic of Imam Khomeini hospital.

### Endpoints

The primary outcomes were to measure asthma clinical symptoms (i.e., shortness of breath during the day and night time, activity limitation, frequency of salbutamol spray consumption and waking up due to asthma symptoms) and asthma severity at the baseline and post-intervention. Secondary outcomes were to measure eosinophils, basophils, blood pressure and lipid profiles pre- and post-intervention.

### Asthma clinical symptoms and severity score

Diagnosis of the allergic asthma was done through measuring the serum levels of immunoglobulin E (IgE) total by IgE enzyme-linked immunosorbent assay (ELISA) kit (DiaMetra DKO060 ELISA Kit, Italy) and the values over than 30 international units are known as allergic patients [2]. Clinical symptoms of the patients were assessed based on the symptoms used as criteria for asthma control [2]. Asthma severity was diagnosed according to the forced expiratory volume in 1 s (FEV1) and clinical symptoms. Those patients with moderate asthma had FEV1 between 60 to 80%, while the ones with mild asthma had FEV1 ≥ 80% and standard clinical symptoms according to GINA [2]. Asthma severity and its clinical symptoms were evaluated at the baseline and endpoint of the trial.

### Collection of blood samples

Blood samples were collected at the baseline and at the end of the study. Each time, 5 mL of blood sample was drawn. The serum samples were frozen at − 20 °C immediately, and were then stored at − 80 °C until further laboratory analyses were done. Blood samples of each patient were collected in the morning after a 12-h fasting, and then the haemolyzed samples were excluded from each analysis and in these cases blood sampling were repeated.

### Laboratory analyses

We used ELISA, as a validated method, (BIO TEK ELX 800 (microplate reader), Highland Park Winooski, VT 05404–0998, USA) for measuring IgE total. During each laboratory analysis, the technicians were blind to the saffron–placebo status. Eosinophils and basophils were evaluated in the results of Complete Blood Count (CBC)-diff. For CBC analysis, 2 ml whole blood in the tubes containing anticoagulant EDTA were placed in the automatic counting machine (Convergy® X5 made in Germany). Lipid profiles (triglyceride, total cholesterol, LDL-C and HDL-C) were measured by enzymatic method kit (Pars Azmoon Co, Tehran, Iran). All laboratory tests were done in the Central Laboratory at Ahvaz Jundishapur University of Medical Sciences.

### Dietary analysis and blood pressure

We assessed the intake of calorie and the percentage of calorie from carbohydrate, protein and fat in all the patients at the baseline and at the endpoint of the trial by 24-h food recalls for two work days and one weekend. The diets were analyzed by the modified nutritionist 4 (NUT4) software. We assessed the calori intake because can affect weight gain and weight gain can affect asthma symptoms and percentage of calori intake from fat is important in respiratory disease because fats have lower work load on respiratory system. Blood pressure was measured in standard method with mercury barometer and after 15 min rest.

### High performance liquid chromatography (HPLC)

The amount of crocin in saffron samples was measured via high performance liquid chromatography (HPLC) method [13]. Following this procedure, 10 mg saffron and 10 ml ethanol 80% were extracted over 15 min sonication in darkness. After that, the samples were centrifuged and, then the supernatant was used for HPLC.

HPLC instrument was done using Agilent technology (1260 infinity II, a multiple wavelength Uv-visible, PDA 1260 infinity model). The column used for separation was ZORBAX Eclipse plus C 18 (Rapid Resolution 4.6 × 100 mm 3.5-Microns). All the experiments were done at room temperature, and the mobile phase composition included a mixture of formic acid (0.2%), acetonitrile and methanol. Linear gradient solution system was used as 76.5% formic acid (0.2%), 13.5% acetonitrile and 10% methanol which changed into 100% methanol by 60 min. The detector was adjusted at different maximum wavelengths including 250 nm, 308 nm and 440 nm. The amount of crocin in the saffron samples was found to be 304.3 mg/kg.

### Statistical analysis

All analyses were done on the intention-to-treat population corresponding to the subjects having consumed at least one dose of the treatment. The normal distribution of the data was assessed using a Kolmogorov–Smirnov test. Blood pressure, lipid profiles, eosinophil and basophil count and dietary factors were analyzed by paired t-test or Wilcoxon paired rank test (when data were not normally distributed) for the within-group comparisons (pre-and post-intervention values in each group). On the other hand, comparisons of changes (endpoint minus baseline) after 8 weeks of intervention between the two groups were done by independent t-test or Mann–Whitney U-test (for non-normal distribution data). Data were reported as mean ± SD or median (25th, 75th percentile) for parametric and non-parametric data, respectively. Chi-squared test, or Fisher’s exact test were used for clinical symptoms of asthma and its severity and also for the baseline characteristic data. All statistical analyses were carried out using SPSS version 16 statistical software (SPSS Inc., Chicago, IL, USA). The most important outcomes were presented at 95% confidence intervals (CI). For all the tests, two-sided *p*-values < 0.05 were considered statistically significant.

## Results

**Fig. 1:**
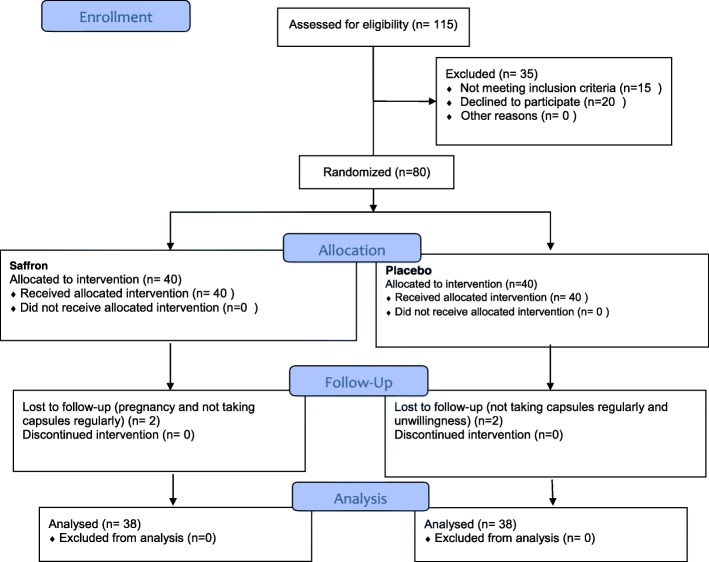
Protocol flow diagram; we conducted a 2 months’ open label, parallel-group, randomized controlled trial to determine the effect of saffron supplementation on the asthma clinical symptoms and asthma severity in patients with mild and moderate persistent allergic asthma

From 80 patients who enrolled the trial, 76 completed the study during the 8-week supplementation period. Among the enrolled ones, 4 discontinued the study (1 subject in the saffron group because of pregnancy, and 1 because of not taking capsules regularly, and in the placebo group 1 was excluded because of not taking capsules regularly, and 1 due to unwillingness). The flow diagram describes the patients’ allocation throughout the study (Fig. 1).

### Baseline characteristics

The mean age of the patients was 41.25 years old (range 18–65 years) and 60% of them were male. In the saffron group, 35% of the patients and in the placebo group 37.5% had mild asthma. Those patients with moderate asthma were 65 and 62.5% in the saffron and placebo groups, respectively. No significant differences were identified in terms of demographic, clinical characteristics, anthropometric, dietary variables, physical activity and asthma severity between the two groups, as indicated in Table 1 (*p* > 0.05). In addition, there were no differences between the intervention and placebo groups regarding the age of onset of asthma at the baseline (Table 1). No significant differences existed in the use of chemical medication (other than asthma drugs) between the trial groups (*p* > 0.05. Data not shown)During the study, there were also no serious adverse events. A few minor feeling of warming up were reported in both groups (but did not lead to discontinuation of the trial on their parts). The drugs consumed by our patients were salbutamol, symbicort, Seroflo and telfast. There was no significant difference between two groups in regard to the drug consumption (*p* = 0.65).

**Table 1:** Comparison of the baseline characteristics between the saffron and placebo groups

Variable	Group		*P*-value
	Saffron *N* = 38	Placebo *N* = 38	
Gender N (%)			
Male N(%)	24(60)	24(60)	0.99
Female N(%)	16 (40)	16(40)	
BMI (kg/m^2^)	26.84 (25.46–27.23)	26.84 (25.05–27.39)	0.79
Age (years)	41.27 ± 9.77	40.77 ± 10.07	0.67
Asthma severity			
Mild N(%)	14 (35)	15 (37.5)	0.81
Moderate N(%)	26 (65)	25 (62.5)	
Systolic pressure (mm Hg)	110.00 (110.00–120.00)	110.00 (110.00–120.00)	0.15
Diastolic pressure (mm Hg)	80.00 (70.00–80.00)	80.00 (70.00–80.00)	0.85
LDL-C (mg/dl)	118.54 ± 36.03	120.74 ± 27.35	0.76
HDL-C (mg/dl)	45.32 ± 13.46	45.57 ± 13.01	0.93
TG (mg/dl)	159.83 ± 41.20	149.30 ± 48.27	0.31
Total-C (mg/dl)	196.82 ± 42.11	192.74 ± 28.93	0.62
Eosinophil (%)	5.60 (3.00–7.45)	4.90 (3.85–6.50)	0.54
Basophil (%)	0.6 (0.3–1.05)	0.6 (0.4–1.20)	0.53
Physical activity (MET-min/week)	676.50 (231–2704)	693 (231–3446.50)	0.71
Symptoms (frequency per day)			
Shortness of breath during the day	1.00 (0.03–1.50)	1.00 (0.04–2.00)	0.73
Shortness of breath during the night	0.71 (0.00–1.87)	0.20 (0.00–1.87)	0.64
Waking up due to asthma symptoms	1.00 (0.00–1.00)	0.00 (0.00–1.00)	0.12
Activity Limitation	0.38 (0.14–1.00)	1.00 (0.10–1.87)	0.22
Salbutamol spray consumption	1.25 (0.02–2.00)	1.00 (0.33–2.00)	0.79
Infant Feeding N (%)			
Breast feeding	30 (75)	32 (80)	
Milk Powder	5 (12.5)	4 (10)	0.37
Breast feeding and milk powder	5 (12.5)	4 (10)	
The age of the onset of asthma (years)	33.50 (23.50–42.50)	30 (25.00–39.00)	0.96

*BMI* body mass index; anti-HSP, LDL-C: low density lipoprotein, *HDL-C* high density lipoprotein, *TG* Triglyceride. Values are expressed as mean ± SD, median ± IQR or number and percent. Mann–Whitney and independent sample T-test were used to compare non-parametric and parametric variables between the two groups, respectively

### Energy intake, physical activity and BMI

Energy intake and calorie percentage from carbohydrate and fat were not significantly different between the saffron and placebo groups (*p* = 0.23, 0.20 and 0.40, respectively). Calorie percentage from protein was statistically significant between the two groups, however the effect of the observed difference was removed with ANCOVA (Table 2). Also physical activity and BMI changes were not significantly different between the groups during the study (*p* = 0.28) (Table 2).

**Table 2:** Within- and between-group comparisons of the changes from baseline to endpoint measures for BMI, physical activity and some dietary intakes in the saffron and placebo groups of asthmatic patients

Variables	Saffron (*N* = 38)		Placebo(*N* = 38)		*P*-value^*^
	Value		Value	*P*-value¥	
BMI (kg/m^2^)					
Baseline	26.84 (25.46–27.23)		26.84 (25.05–27.39)		
8 weeks (endpoint)	26.68(25.81–27.18)		26.84 (24.79–27.44)	0.37	0.28
Changes	0.00 (−0.35) to (0.00)		0.00 (0.00–0.32)		
Physical activity (MET-min/week)					
Baseline	676.50 (231–2704)		693 (231–3446.50)		
8 weeks (endpoint)	660.00 (231.00–2220.50)	0.56	693.00 (379.50–3876.00)	0.02	0.28
Changes	0.00 (0.00–90.75)		0.00 (0.00–173.250)		
Energy intake (kilocalories)					
Baseline	1858.97 ± 213.16		1856.47 ± 99.66		
8 weeks (endpoint)	1865.36 ± 208.48	0.46	1877.44 ± 112.05	0.02	0.23
Changes	32.00 (−41.00) to (46.25)		31.00 (−30.00) to (62.00)		
Percent of calories from carbohydrate (%)					
Baseline	54.11 ± 6.63		57.15 ± 5.19		
8 weeks (endpoint)	54.65 ± 6.65	0.81	56.61 ± 6.36	0.16	0.20
Changes	0.24 (−1.30) to (1.87)		0.21 (−2.21) to (0.77)		
Percent of calories from protein (%)					
Baseline	11.69 ± 1.54		11.95 ± 1.51		
8 weeks (endpoint)	11.81 ± 1.46		11.81 ± 1.33		0.03
Changes	0.13 (−0.24) to (0.47)	0.16	− 0.07 (− 0.47) to (0.12)	0.11	
Percent of calories from fat (%)					
Baseline	33.80 ± 4.36		35.73 ± 6.03		
8 weeks (endpoint)	33.79 ± 4.35		36.47 ± 6.51		0.40
Changes	0.23 (−1.97) to (2.02)	0.87	0.14 (−0.69) to (1.83)	0.21	

*BMI* Body Mass Index. *p-value for comparing the changes of variables between the groups. Mann-whitney test was used. ¥*p*-value for comparing baseline, with endpoint values within each group. Wilcoxon and Paired t test were used. Values are expressed as mean ± SD for parametric and median (25th, 75th percentiles) for non-parametric data

### Asthma symptoms and asthma severity

Asthma clinical symptoms including shortness of breath during the day and night time, activity limitation due to asthma, waking up due to asthma symptoms and frequency of spray consumption improved in the saffron group in comparison to the placebo group (*p* < 0.001). Besides, asthma severity improved in the saffron group (18.40% reduction in asthma severity in the saffron group in comparison to 2.60% in the placebo group), but there were no significant differences with regard to the mean change of severity of asthma between the two groups (*p* = 0.076) (Table 3).

**Table 3:** Within-and between-group comparisons of the changes from baseline to endpoint measures for clinical symptoms, basophils, eosinophils and asthma severity in the saffron and placebo groups of asthmatic patients

Symptoms (frequency per day)	Saffron (*N* = 38)	Placebo(*N* = 38)	*P*-value^*^
Shortness of breath during the night			
Baseline	0.71 (0.00–1.87)	0.20 (0.00–1.87)	
8 weeks (endpoint)	0.00 (0.00–1.00)	0.02 (0.00–1.62)	< 0.001
Changes	−0.24 (− 1.00–0.00)	0.00 (0.00–0.00)	
*P*-value^¥^	< 0.001	0.02	
Waking up due to asthma symptoms			
Baseline	1.00 (0.00–1.00)	0.00 (0.00–1.00)	
8 weeks (endpoint)	0.00 (0.00–0.00)	0.00 (0.00–1.00)	< 0.001
Changes	−0.50 (− 1.00–0.00)	0.00 (0.00–0.00)	
*P*-value^¥^	< 0.001	0.02	
Activity Limitation			
Baseline	0.38 (0.14–1.00)	1.00 (0.10–1.87)	
8 weeks (endpoint)	0.03 (0.000–1.00)	1.00 (0.06–2.00)	< 0.001
Changes	−0.14 (−0.79–0.00)	0.00 (0.00–0.00)	
*P*-value^¥^	< 0.001	0.49	
Salbutamol spray consumption			
Baseline	1.25 (0.02–2.00)	1.00 (0.33–2.00)	
8 weeks (endpoint)	0.14 (0.00–1.00)	1.00 (0.38–2.00)	< 0.001
Changes	−0.50 (−1.00–0.00)	0.00 (0.00–0.00)	
*P*-value^¥^	< 0.001	0.24	
Eosinophil (%)			
Baseline	5.60 (3.00–7.45)	4.90 (3.85–6.50)	
8 weeks (endpoint)	4.55 (2.82–6.00)	4.90 (2.97–6.00)	0.12
Changes	−0.85 (−1.62) to (0.15)	0.05 (−1.62) to (0.57)	
*P*-value^¥^	0.005	0.58	
Basophil			
Baseline	0.60 (0.3–1.05)	0.60 (0.4–1.20)	
8 weeks (endpoint)	0.90 (0.50–1.50)	1.10 (0.77–1.50)	0.09
Changes	0.00 (−0.20) to (0.62)	0.40 (0.00–0.80)	
*P*-value^¥^	0.06	< 0.001	
Asthma severity changes (%)			
Decrease	18.40	2.60	
No change	78.90	92.10	0.076
Increase	2.6	5.3	
Asthma severity baseline N(%)			
Mild	14(35)	15 (37.5)	0.81
Moderate	26 (65)	25 (62.5)	
Asthma severity endpoint N(%)			
Mild	20 (52.60)	14 (36.80)	0.16
Moderate	18 (47.40)	24 (63.20)	

*p-value for comparing the changes of variables between the groups. Mann-whitney test was used. ¥*p*-value for comparing baseline, with endpoint values within each group. Wilcoxon test was used. Values are expressed as median (25th, 75th percentiles) (non-parametric data)

### Eosinophil and basophil count

Eosinophils significantly decreased in the saffron group (*p* = 0.005), however the changes were not significant in the placebo group (*p* = 0.58) and between the groups (*p* = 0.12). Basophil count significantly increased in the placebo group (*p* < 0.001), but the changes in the saffron group and also between the two groups were not significant (*p* = 0.06 and 0.09, respectively) (Table 3).

### Blood pressure and lipid profile

Changes in systolic blood pressure during the study was significant in the saffron group as compared to the placebo. This shows that saffron reduced the systolic blood pressure (*p* = 0.03). It also decreased diastolic blood pressure in comparison to the placebo group (*p* = .04). Besides, Triglyceride and LDL-C levels in the saffron group decreased significantly, when compared to those in the placebo (*p* = 0.002 and < 0.001 respectively). Saffron also decreased total cholesterol and increased HDL-C, however the changes were not significant between the two groups (*p* = 0.08 and 0.68, respectively) (Table 4).

**Table 4:** Within- and between-group comparisons of the changes from baseline to endpoint measures for blood pressure and lipid profile in the saffron and placebo groups of asthmatic patients

Variables	Saffron (*N* = 38)		Placebo(*N* = 38)		*P*-value^*^
	Value	*P*-value^¥^	Value	*P*-value¥	
Systolic pressure (mm Hg)					
Baseline	11.00 (11.00–12.00)		11.00 (11.00–12.00)		
8 weeks (endpoint)	11 (10.75–12.00)		11.75 (11.00–12.00)		
Changes	−0.11 ± 0.39	0.08	0.02 ± 0.11	0.70	0.03
Diastolic pressure (mm Hg)					
Baseline	8.00 (7.00–8.00)		8.00 (7.00–8.00)		
8 weeks (endpoint)	7.25 (7.00–8.00)		8.00 (7.50–8.00)		
Changes	−0.02 ± 0.44	0.15	0.15 ± 0.30	0.005	0.04
Total cholesterol					
Baseline	196.82 ± 42.11		192.74 ± 28.93		
8 weeks (endpoint)	162.52 ± 36.95		173.47 ± 39.87		0.08
Changes	−34.28 ± 32.23	< 0.001	−19.26 ± 42.04	0.008	
Triglyceride					
Baseline	159.83 ± 41.20		149.30 ± 48.27		
8 weeks (endpoint)	139.48 ± 34.39		153.18 ± 44.99		0.002
Changes	−20.34 ± 30.28	< 0.001	3.88 ± 34.94	0.49	
LDL-C					
Baseline	118.54 ± 36.03		120.74 ± 27.35		
8 weeks (endpoint)	80.09 ± 35.85		121.82 ± 35.16		< 0.001
Changes	−38.44 ± 41.82	< 0.001	1.08 ± 25.76	0.79	
HDL-C					
Baseline	45.32 ± 13.46		45.57 ± 13.01		
8 weeks (endpoint)	48.34 ± 15.49		47.35 ± 10.62		0.68
Changes	3.02 ± 15.53	0.23	1.78 ± 10.51	0.30	

*LDL-C* low density lipoprotein, *HDL-C* high density lipoprotein *p-value for comparing the changes of variables between the groups. Mann whitney test was used for systolic and diastolic blood pressure and independent sample T test for lipid profile. ¥*p*-value for comparing baseline, with endpoint values within each group. Paired samples t-test was used. Values are expressed as mean ± SD for parametric data and median (25th, 75th percentiles) for non-parametric data

### Crocin measurement

Results of HPLC method showed that the amount of crocin in the saffron sample was 304.3 mg/kg We measured this for sample preparation to ensure consistency of crocin amount in saffron.

## Discussion

This study was designed to assess the effect of saffron on patients with allergic asthma in a randomized double blind clinical trial. The findings indicated that saffron supplement therapy has beneficial effects on asthma severity and improved its clinical symptoms including shortness of breath during the day and night time, use of salbutamol spray, waking up due to asthma symptoms as well as activity limitation and overall asthma symptoms, as compared to the placebo group.

A research conducted on healthy humans suggested that doses up to 400 mg per day saffron are safe [14]. Similarly, Verma and Bordia (1998) showed that 100 mg saffron supplementation improved the anti-oxidant status in patients with coronary heart disease [15]. Also, a recent study reported that 100 mg saffron in patients with metabolic syndrome reduced anti-heat shock protein 70 (which is a novel risk factor for asthma and is correlated with asthma severity [16, 17]. So according to previous studies the dos we used in this trial (100 mg) is safe and effective. To our best knowledge, this is the first clinical trial that has assessed the effect of saffron supplementation on allergic asthmatic patients.

Generally, in the process of airway inflammation in asthma, a variety of cells such as lymphocytes, eosinophils, mast cells, and neutrophils are involved. This inflammation can, in turn, lead to recurrent episodes of breathlessness, wheezing, chest tightness, and coughing in the susceptible individuals. The major inflammatory cells involved in the pathophysiology of asthma are eosinophils which their maturation, activation, survival, and recruitment within the airway lumen are key pathogenic events in the development of allergic and also non-allergic asthmatic phenotypes. Additionally, complex proinflammatory and immunologic mechanisms are involved in eosinophilic asthma, mainly T helper (Th)2 lymphocytes which release interleukins (IL- 5, IL-4, and IL-13) [18].

Generally, according to the previous studies on animal models of allergic airway disease,saffron has several beneficial effects on asthma; such as anti-inflammatory [9, 11, 19], antioxidant [15, 20], immunomodulatory [21] and muscle relaxing [10] effects. Also we reported that saffron in asthmatic patients reduced anti-heat shock protein 70 (as a novel risk factor for asthma severity) and high sensitive-c reactive protein and also improved the lung function (assessed via spirometry test) [22]. Accordingly, saffron is believed to improve the asthma symptoms due to airway inflammation (anti-inflammation), hyper-responsiveness (immunomodulation) and muscle contraction (muscle relaxation). Overall, these processes may indicate the effectiveness of saffron in improving asthma symptoms and severity, as reflected in the results of the present study. Saffron has beneficial therapeutic effects on respiratory diseases, as well. Boskabady et al., (2006) reported that saffron has relaxant effects on guinea-pig tracheal chains [10] This, in turn, may be one of the reasons showing that how saffron improved the asthma symptoms in our study. Some possible mechanisms of the relaxing effects of saffron include the inhibition of histamine H1, muscarinic receptors and calcium channels, the activation of ß2-adrenoceptors and also the modulation of nitric oxide (NO) [23].

Gholamnezhad et al., (2013) showed that the saffron extract decreased serum levels of endothelin 1(as an inflammatory marker) in sensitized guinea pig (animal models of asthma) [11]. These findings are in line with our results showing that saffron improved some lipid profile factors and blood pressure. Similarly, Shirali et al., found that crocin supplementation decreased triglyceride, total cholesterol and LDL-C, and also increased HDL-C in type 2 diabetic rats [12].

## Conclusions

In conclusion our findings showed the beneficial effects of 100 mg saffron daily on asthma symptoms, severity, and also on systolic and diastolic blood pressure, triglyceride and LDL-C.. As our trial is the first study investigating the effects of saffron on asthma in human models; thus more studies with longer supplementation periods are required to provide additional evidence supporting the use of saffron in clinical settings.

## Abbreviations

Anti-HSP: Anti-heat shock protein
BMI: Body mass index
CRP: C-reactive protein
FEF: Forced expiratory flow
FEV1: Forced expiratory volume in 1 s
FVC: Forced vital capacity
HDL-C: High density lipoprotein
ICS: Inhaled corticosteroid
IPAQ: International physical activity questionnaire
LDL-C: low density lipoprotein
